# The clinical practice guideline palliative care for children and other strategies to enhance shared decision-making in pediatric palliative care; pediatricians’ critical reflections

**DOI:** 10.1186/s12887-019-1849-0

**Published:** 2019-11-29

**Authors:** Dunja Dreesens, Lotte Veul, Jonne Westermann, Nicole Wijnands, Leontien Kremer, Trudy van der Weijden, Eduard Verhagen

**Affiliations:** 1 Department of Family Medicine, Maastricht University/School CAPHRI, P.O. Box 6166200, MD Maastricht, the Netherlands; 2 Knowledge Institute of the Federation of Medical Specialists, Utrecht, the Netherlands; 3 GGD-regio Utrecht, Utrecht, the Netherlands; 4 GGzCentraal, Amersfoort, the Netherlands; 5 Maastricht University Medical Centre+, Maastricht, the Netherlands; 6 Department of Pediatrics, Emma Children’s Hospital/Amsterdam UMC, Amsterdam, the Netherlands; 7 Princess Maxima Centre, Utrecht, the Netherlands; 8 University of Groningen, Groningen, the Netherlands; 9 Beatrix Children’s Hospital/University Medical Centre Groningen, Groningen, the Netherlands

**Keywords:** Clinical practice guideline, Shared decision-making, Palliative care, Children, Interviews, Qualitative

## Abstract

**Background:**

Because of practice variation and new developments in palliative pediatric care, the Dutch Association of Pediatrics decided to develop the clinical practice guideline (CPG) palliative care for children. With this guideline, the association also wanted to precipitate an attitude shift towards shared decision-making (SDM) and therefore integrated SDM in the CPG Palliative care for children. The aim was to gain insight if integrating SDM in CPGs can potentially encourage pediatricians to practice SDM. Its objectives were to explore pediatricians’ attitudes and thoughts regarding (1) recommendations on SDM in CPGs in general and the guideline Palliative care for children specifically; (2) other SDM enhancing strategies or tools linked to CPGs.

**Methods:**

Semi-structured face-to-face interviews. Pediatricians (15) were recruited through purposive sampling in three university-based pediatric centers in the Netherlands. The interviews were audio-recorded and transcribed verbatim, coded by at least two authors and analyzed with NVivo.

**Results:**

Some pediatricians considered SDM a skill or attitude that cannot be addressed by clinical practice guidelines. According to others, however, clinical practice guidelines could enhance SDM. In case of the guideline Palliative care for children, the recommendations needed to focus more on how to practice SDM, and offer more detailed recommendations, preferring a recommendation stating multiple options. Most interviewed pediatricians felt that patient decisions aids were beneficial to patients, and could ensure that all topics relevant to the patient are covered, even topics the pediatrician might not consider him or herself, or deems less important. Regardless of the perceived benefit, some pediatricians preferred providing the information themselves instead of using a patient decision aid.

**Conclusions:**

For clinical practice guidelines to potentially enhance SDM, guideline developers should avoid blanket recommendations in the case of preference sensitive choices, and SDM should not be limited to recommendations on non-treatment decisions. Furthermore, preference sensitive recommendations are preferably linked with patient decision aids.

## Background

It is estimated that 750–1000 children with a life-threatening illness die each year in the Netherlands and 5000 children are diagnosed with a life-threatening illness [1]. Of the 1200 Dutch pediatricians, 60 pediatricians (being part of pediatric palliative teams in university medical centers) regularly encounter children with a life-threatening disease. Most Dutch pediatricians – and other healthcare professionals – infrequently encounter children who are in need of palliative care, making them less sure how to proceed in these cases. Moreover, a life-threatening illness has an enormous impact on both the parents and the child. The pediatrician can help in coping, and counseling on treatment related issues as well psycho-social aspects should start as early as possible. Furthermore, new developments in pediatric palliative care and limited knowledge thereof among pediatricians led to unwarranted practice variation [1, 2]. The Dutch Association of Pediatricians therefore deemed it necessary to develop the clinical practice guideline (CPG) Palliative care for children to improve the quality of care for these children. The objective of this guideline is to provide pediatricians support and guidance when caring for children with a life-threatening disease. The guideline was published in the summer of 2013 [3].

The introduction of the CPG Palliative care for children stresses the importance of knowing and discussing the needs of the patient and his or her parents. In addition to palliative intervention recommendations, the guideline consequently also contains recommendations on communication and shared decision-making (SDM) (see Table 1) [3]. The underlying assumption of SDM models is that the doctor-patient relationship is based on mutual respect and on a joint interest to achieve the most beneficial outcomes for the patient, with partners sharing decisions. In this process it is important to understand the patients’ preferences and values, and to acknowledge the doctor’s expertise [4]. However, it is still unclear if and how SDM can be incorporated in CPGs [5–9]. Van der Weijden et al. describe how guidelines can be adapted to facilitate SDM, e.g. by incorporating patient-centered questions, flagging recommendations for which considering patient preferences is particularly important, or by embedding relevant patient support tools [5]. The Institute of Medicine recommends including patients in the guideline panels to address heterogeneity in patient preferences and include trade-offs in recommendations [10]. Prior to 2013, only a few guidelines tried to do this [11–13].

**Table 1:** Quick overview of the CPG Palliative care for children

Clinical practice guideline Palliative care for children (NVK, 2013)	
The guideline consists of three chapters, each providing recommendations on the topic at hand.	
(1) Symptoms	
Recommendations on recognition and treatment of symptoms, such as anxiety, nausea, pain, spasticity and fatigue.	
(2) Communication & decision-making	
Recommendations on decision-making, such as:	
- Take into account the values and preferences of the child and/or parents;	
- Be conscious of the fact that decision-making is a continuous process;	
- Ask the child and/or parents at various moments which decision-making role they would like to have;	
- Carry out the conversations in a quiet, private and comfortable space;	
- Clarify mutual goals;	
- Confer with the child and the parents, […], use nonprofessional language, and repeat what has been discussed;	
- Record the view of the parents in the patient file.	
(3) Organisation of palliative care	
Recommendations on the organisation of care, such as coordination & responsibilities, patient file, educating healthcare professionals and financing palliative care for children.	

Sharing decisions in pediatric care is triadic in nature as parents or other care givers may be as much or even more involved as the child [14]. Furthermore, in the Netherlands children have a right to be involved in deciding about their care from the age of 12 years [15]. A majority of both parents and children prefer to participate in treatment decision-making and consider it an essential component of quality of care, together with relationship building, demonstration of effort and information exchange [16, 17]. Despite the large number of publications in support of children’s (ethical) right – and desire – to participate in their own health care and decisions, pediatricians usually do not involve them in the decision-making. One reason being the clinicians’ underestimation of children’s capabilities; because of the children’s age, pediatricians think they cannot or do not want to be involved in the decision making [18, 19]. A national survey (2011) among Dutch pediatricians showed that 21% perceived a need to share the final responsibility for an end-of-life decision with the parents. Half of the respondents would inform parents and ask for their permission to discontinue treatment, and a quarter would merely inform parents [20]*.* Reasons for not sharing the decision were: complexity/uncertainty of the decision at hand, and/or protecting the parents. Another important aim of the guideline Palliative care for children was therefore to trigger an attitude shift towards SDM in palliative pediatric care.

The aim was to gain insight if integrating SDM in CPGs can potentially encourage pediatricians to practice SDM. Its objectives were to explore pediatricians’ attitudes and thoughts regarding (1) recommendations on SDM in CPGs in general and the guideline Palliative care for children specifically; (2) other SDM enhancing strategies or tools linked to CPGs.

## Methods

We applied the Standards for reporting qualitative research (SRQR) and Consolidated criteria for reporting qualitative studies (COREQ) for this article [21, 22].

### Study design

We conducted semi-structured, face-to-face interviews to gather ‘deep’ information and perceptions of the participating pediatricians using a phenomenological approach [23, 24]. This qualitative research method emphasizes the importance of personal perspective and interpretation. It is a suitable method for gaining insight into participants’ motivations and actions and understanding subjective experience.

### Researchers’ characteristics

The second author (LV) conducted the face-to-face interviews, with the assistance of JW and NW. LV, NW and JW were sixth year medicine students carrying out a science research internship at Maastricht University. There were no prior relationships between the participants and any of the authors. We informed the participants of the researchers’ background and their roles in the PhD-research of the first author (DD), (1) at the time of recruitment, and (2) at the start of the interview. The interviewer (LV) had no previous experience with interviewing. She was trained on the job by TVDW and DD and closely supervised by the research team through regular meetings and feedback on audio-recorded interviews.

### Participants

We purposively sampled pediatricians with experience in providing palliative pediatric care. The respondents were also purposeful sampled regarding tenure (professor, specialist, fellow); age; and gender. We recruited pediatricians from five subspecialty divisions: oncology, metabolic diseases, neurology, intensive care and neonatology at three university based pediatric centers in the Netherlands. These centers were UMCG, MUMC+ and Amsterdam UMC location AMC. We tailored the approach to each pediatric center to ensure timely recruitment of participants. Participants were approached via personal mailings sent by the author linked to the specific pediatric center or via personal invitations by one of the authors (LK, EV). The interviews took place at an office in the hospital in the second quarter of 2014. During the interview, only the participant and interviewer were present.

### Data collection

Based on the relevant literature and the guideline Palliative care for children (PCFC), an interview guide was developed [25]. Table 1 contains an overview of the (recommendations of the) guideline. The research team discussed the interview guide within the project team and the guide was piloted in two interviews. We followed an iterative approach; meaning that the interview guide was adjusted – if needed – in response to the preceding interviews (see Additional file 2).

During the pilot interviews, it became clear that not all participants might have an in-depth knowledge of the guideline PCFC. Consequently, it was decided that if the pediatrician was not familiar in detail with the guideline that the researcher would walk the pediatrician through the guideline and the SDM-recommendations at the start of the interview.

In addition to the PCFC guideline, all participants were presented with two other SDM-approaches [5]. First, participants were shown two patient decision aids (PDA) for children: one on anticoagulants and another on bone infection [26, 27]. Second, we showed them the PCFC guideline recommendation on pain relief (see Additional file 1) that we had rewritten so that it offered several options for pain relief and could stimulate discussion with the patient and/or parents. Approval for the study protocol was granted by the Medical Ethical Committee of the University Medical Center Groningen, reference number M14.150.681/METc 2014.057 (10 February 2014).

Verbal informed consent was gained from all participants, which included consent for use of anonymized quotes in publications. No follow-up interviews took place. The interviews were audio-recorded and transcribed verbatim by LV, JW and NW, with at least one author carrying out a reliability check on each transcript correcting any transcribing errors. The participants received the transcripts for comments, correction and approval (member check). Furthermore, LV made field notes before and after the interviews, and these were discussed with DD.

### Data analysis

Data analysis was performed by using the constant comparative method [23]. Three authors (LV, NW and DD) read and reread all transcripts independently. Text fragments related to the research questions were selected and coded. The research team did not identify themes or codes in advance.

The codes were developed inductively, while reading the transcripts and making notes about selected fragments in the transcripts. After the first five transcripts, the researchers (LV, DD) compared their observations and developed preliminary coding categories. After analyzing individual interviews, we added and altered codes and categories in an iterative process. LV and DD discussed disagreement about the coding until consensus was reached. When all the interviews were analyzed and coded, we collected the codes in a preliminary coding scheme. Through axial analysis, we constructed (sub-) categories. LV and DD discussed and compared all the codes and categories resulting in the final coding scheme (see Additional file 3). Data analysis was supported by NVivo-software. The research team met to discuss and complete the study report and manuscript. We did not invite participants to provide feedback on the findings.

## Results

### Sample

In total 22 pediatricians were approached to be interviewed and 15 agreed to participate (Table 2). Three were moderately interested and preferred to decline, three did not respond and one pediatrician was not interested in the subject and declined. The participants consisted of two professors, 12 medical specialists, and one fellow. Nine of them had 15 years of experience or more.

**Table 2:** Characteristics of the interviewed participants

Interview # per specialism	Gender	Hospital code	Duration of interview (minutes)
Oncology			
1	M	1	58
10	F	3	75
11	M	2	43
15	M	3	34
Intensive care			
8	F	1	30
9	M	2	60
Metabolic disease			
3	M	1	54
6	M	1	47
12	M	3	39
14	F	3	20
Neonatology			
2	F	1	65
5	M	1	46
13	M	2	48
Neurology			
4	F	1	45
7	M	1	57

*F* Female.

*M* Male.

### Data collection

The 15 interviews lasted between 20 and 75 min (average 48 min). Data saturation occurred after eight interviews; no new codes emerged from the data.

### Main findings

Three main findings became apparent when analyzing the interviews.

1. Possibility (or desirability) of CPGs enhancing the practice of SDM;
2. Added value of integrating SDM in CPGs in general and the PCFC guideline specifically;
3. Usefulness of a PDA accompanying a guideline.

Before describing the main findings, it is important to note that interviewees interpreted the term SDM differently. When asked to describe SDM, half of the participants referred to it as reaching a decision together with other clinicians, e.g. during a (multidisciplinary) team meeting without involving the patient. After providing participants with a commonly used definition of SDM, and briefly discussing the concept, several participants were of the opinion that if there was a clear superior treatment option, the decision should lie with the clinician and there was no need for patient involvement. Almost half of the participants found that patients and/or parents needed to be included only when there was not an obvious best choice (“grey area”), or when decisions were not treatment related, e.g. decisions about where to die or how to say their goodbyes. Furthermore, there seemed to be confusion between sharing decisions and who has the final responsibility for the decisions made. Some participants did not want to burden the patient/parents with the responsibility of deciding on their child’s (end-of-life) care, stating: *“The parents have to live with that decision.”* (Participant 3).

### Possibility (or desirability) of CPGs enhancing the practice of SDM

When asked if CPGs could potentially enhance SDM, the responses were mixed. About half of the participants mentioned that it could be helpful when there is disagreement; to make sure you cover all the relevant topics, including topics you yourself might consider less important, but are valuable to patients; and to ensure completeness of information provided.

Some pediatricians commented that CPGs and SDM do not go together as they regarded CPGs paternalistic in principle and are therefore irreconcilable with SDM. Others thought it could be a good combination (Table 3).

**Table 3:** Illustrative quotes on possibility (or desirability) of CPGs enhancing the practice of SDM

Theme:	Possibility (or desirability) of CPGs encouraging the practice of SDM *Illustrative quote*	Interview #
Useful/helpful		
	*“Perhaps it could help a clinician who is SDM-minded, but doesn’t dare. I mean, if you aren’t experienced in palliative care yet, it can be quite, um, scary.”*	3
	*“Sure, especially when it comes down to points of disagreement, it can be helpful to choose a certain direction. In other guidelines, it is mentioned explicitly that you need to check with other specialists before you proceed. And with the patient and parents, of course.*”	4
	*“For some aspects, it could be the case that you yourself don’t consider them, or that you are partial to certain things. But by mentioning all the aspects in a structured way in the guideline and discussing these with the patients, all topics that matter are covered.”*	12
	*“If you go through a list the patient probably thinks that you are a meticulous doctor who makes sure to tick all the boxes. However, it can also appear as being callous because it is not a conversation, you just go through the list.”*	3
	*“It must be useful when your peers have thought about it and decided to add recommendations on SDM, to support you or make it easier for you to do it yourself.”*	11
	*“It means you can adhere to the guideline more often without you having to explain time and again why you didn’t adhere.”*	14
Guidelines are paternalistic		
	*“A guideline will not immediately contribute to SDM. SDM depends on communication. And a guideline can obstruct that, as it says ‘do this’ and some people will claim that they are legally required to follow the guideline.”*	13
	*“Often guidelines say do A, B or C. They don’t say consider A, B or C because there’s the same amount of evidence. And if it would say consider A, B or C then you can discuss it with the parents.”*	1
SDM is an attitude/skill		
	*“You don’t need a guideline on how to have a conversation.”*	9
	*“If doctors don’t do SDM, a guideline will not make them do SDM.”*	1
	*“I think that the way you involve the parents is ‘the art of medicine’ and not medicine. So, I think it comes down to the competencies of the doctor. You can’t capture those in a guideline.”*	2
SDM irrespective of guideline subject		
	*“I don’t think it will help to integrate SDM in the guideline. A guideline helps when there’s no common sense. This is common sense.”*	10
	*“Maybe we need a guideline on SDM; namely involve the parents and the patient with every choice concerning their care. These two lines, easy as that.”*	1

Another reason why some participants said that CPGs and SDM do not go together was because they regarded SDM an attitude or skill. A pediatrician is either willing or capable to share decisions with a patient or not, and adding SDM as a recommendation to a guideline will not change that attitude. *“It is the difference between ‘the art of medicine’ and ‘medicine’ “*, according to two participants (2 and 12). Several participants commented that to support SDM as a skill, the recommendations in the CPG needed to be more practical. For example: one of the guideline recommendations is to clarify and collaboratively set goals; but no practical guidance is offered on how to do this in clinical practice. Some participants, however, thought that incorporating recommendations on SDM in a CPG, might make it easier to practice it because e.g. it makes it less scary (Table 3).

### SDM integrated in guidelines

Several options exist to address SDM in a guideline. The PCFC guideline opted to add recommendations on (shared) decision-making in the guideline as a separate chapter (see Table 1). The participants were asked how they felt about the innovative section on decision-making in the guideline. A participant noted that the recommendations on (shared) decision-making could help prepare the discussions with the patient/parents, and could help to put the patient in a social context. Several of the participants thought that most of the SDM-recommendations stated the obvious and that experienced pediatricians already practiced this. However, others said the recommendations might be useful for pediatricians who are less experienced or a bit hesitant. One participant indicated that the SDM-recommendations are valid for pediatrics in general and therefore need not be in this guideline. He suggested developing a separate guideline solely on SDM in pediatrics (Table 4).

**Table 4:** Illustrative quotes on added value of SDM integrated in CPGs

Theme:	Added value of SDM integrated in the guideline PCFC and guidelines in general *Illustrative quote*	Interview #
SDM-recommendations in guideline PCFC		
	*“I feel that a disadvantage of this guideline is that, even though it is corroborated by evidence, that the recommendations state the obvious.”*	1
	*“If you write it down in such detail, I would like to know the added value for myself. What’s in it for me when I have to deal with a high complex situation? However, do I think everyone applies these SDM-recommendations all the time in practice? No.”*	10
	*“If you are inexperienced, you can read these SDM-recommendations. I think it’s nice, but they are not really practical.”*	3
Modified multi-option (SDM) recommendation		
	*“It’s more agreeable. Also, because it contains the lines of reasoning.”*	6
	*“It provides more information, more possibilities to consider.”*	11
	*“This does represent the actual situation you’re dealing with in practice. Do you as a doctor take the lead, or do you provide more options? So that the parents are in a position to choose as well.”*	7
	*“For the more complex decisions, of which there many in palliative care, it is beneficial to indicate the whole spectrum of care.”*	12
	*“It will probably, even if it’s only subconsciously, prompt you to explain more to the parents, such as harms and alternatives, because it’s right in front of you.”*	5
	*“Nothing is black and white. And then it is nice that the guideline also provides alternatives that meet a certain standard; it prompts you to more discussion on what to do.”*	14
	*“The SDM-recommendation requires more deliberation and demands more of your communication skills.”*	13
	*“I would like to know the evidence base of each item mentioned. If it’s part of the guideline, I would question if it was properly assessed.”*	10
	*“And, there is of course a balance in what makes a guideline practical. You cannot include all the literature, because then it would no longer be a practical guideline.”*	15
	*“If you want you can put everything in a guideline. I don’t believe that’s the way to go.”*	5
	*“Especially for the doctors who are being trained, nurses and physician assistants who are less experienced, have less knowledge, they will need a very clear guideline.”*	13

#### Modifying a recommendation into an SDM-recommendation

When showing the participants, the original single-option recommendation on pain relief and the modified multiple-option recommendation from the symptoms’ section of the PCFC guideline (see Additional file 1), most participants preferred the modified recommendation. The modified recommendation was viewed as beneficial to engage patients/parents in the decision-making by showing considerations that are important to the patient/parents (Table 4). According to one participant the usefulness of the short, single-option recommendation versus the longer, multiple-option recommendation depends on the context, e.g. in acute or neonatal intensive care, the original – shorter – recommendation would be more useful. Views expressed by the participants working in neonatal intensive care reflected this notion.

### Usefulness of a PDA accompanying a guideline

Another option to address SDM in guidelines is paring a specific conditional recommendation with a PDA. A conditional recommendation is used when the underlying evidence is scarce or conflicting, or when more than one relevant treatment options is available. For conditional recommendations, it is known that an individual patient values the uncertainties and trade-offs differently compared to other patients [28]. Although most participants perceived the PDA to be beneficial for the patient/parents, and to a lesser degree for themselves. Most of the participants preferred to convey the information mentioned in the PDA in person, out of fear of losing rapport with the patient when looking at a piece of paper or tablet during a consultation. Others believed patients/parents would not want to use PDAs. Some interviewed pediatricians expressed that the PDAs contained useful topics that they would normally not address during consultation, and suggested it could be used as a checklist to ensure important topics were covered. Two participants mentioned that patients/parents often do not remember everything mentioned during a consultation and that PDAs are ideal for patients as a reference document to take home, re-read and reflect on what was discussed (Table 5).

**Table 5:** Illustrative quotes on usefulness of a PDA accompanying the CPG

Theme:	Usefulness of a PDA accompanying the CPG *Illustrative quote*	Interview #
Usefulness of PDAs to patient/parents		
	*“If the effects* [of the interventions] *are the same, then of course the parents can make the decision. And it’s quite neat to have all the pros and cons on paper.”*	5
	*“I must honestly say, based on what I’m seeing right now, it would be nice to hand them* [patient/parents] *something. If they want to think about it, they can weigh the pros and cons.”*	7
	*“It can indeed be handy to make a decision, because sometimes the practicalities of a treatment decision elude you* [as a doctor]*, but those can be very important to the parents.”*	12
	*“It is easy to have something like that, listing all the points. It won’t make it easier, but it will make it more transparent. We don’t have a lot of those yet, do we?”*	13
	*“You can discuss it and read it again later. And it’s way better than my illegible handwriting.”*	14
	*“The patients remember only 20% of what’s being discussed. And, because you can give it to take home, it means that people don’t need to make on the spot decisions during consultation.”*	14
	*“I’m not sure if these make it much clearer. It could also complicate matters for patients, these kind of choices.”*	7
	*“I think it’s a lot of text. I don’t think patients will read it.”*	9
	*“It’s more of a checklist for myself, not something you go through together.”*	11
Usefulness of PDAs to pediatricians		
	*“When I look at it, I immediately notice that it covers a dilemma we deal with a lot. And it is actually a nice format, so I definitely think it’s of use to us. Can you email it to me?”*	6
	*“I think so. It shows you the experience of others in these instances, what they did. And it also gives you an idea of what to ask in cases you yourself are a bit hesitant about it.”*	11
	*“Sometimes when talking to parents, you notice they get confused, and you need to tell more. If you can show it with visuals like this, is even better.”*	13
	*“Yes, I think it’s really practical: not a lot of text and it looks appealing. Most of it, is in your head, but this makes the considerations really explicit. And translates it directly to aspects patients care about.”*	15
	*“It doesn’t need to be part of a guideline, I can do it myself.”*	1
	*“I’d prefer to explain it myself. It is part of being a doctor. You’ll notice a response, a hesitation. And as a doctor, you prefer some medicines yourself, because you’re more familiar with them and you’ll advise those.”*	4
	*“No, I can do that myself. I’m more inclined, on the basis of my experience and taking the patient into account, to take the lead and say: ‘I think this is the best medicine’.”*	7
	*“I think I can explain it easily in 5 min. The question is whether that is true. Okay, you’re making me reconsider my answer.”*	7
	*“I don’t think I would use it. I feel a bigger urge to look the patient in the eyes and tell them what’s it about.”*	10
	*“I don’t think it’s right. I suppose additional explanations are needed? But, I think they’re risky, because it will affect the verbal communication negatively.”*	13

## Discussion

Several participants acknowledged the added value of SDM being included in the guideline PCFC, and more participants were of the opinion that guidelines in general potentially could enhance SDM. Regarding the specific SDM-recommendations in the PCFC guideline, several participants judged these as stating the obvious and lacking detail and practical guidance. Some even said they were offended by these recommendations, perceiving it as an attack on their professionalism because they already do this. Clinical observations, however, have shown that SDM during patient contacts is not standard practice yet. Clinicians think or say that they practice SDM, but when their interactions with patients are analyzed, it appears that the level of SDM leaves room for improvement [9, 29–32]. The felt attack could perhaps be unjust because of optimistic bias. Furthermore, research suggests that there seems to be a tendency with clinicians to share non-treatment related decisions with patients, and to share the treatment related decisions to a lesser degree [33, 34]. When it concerns children, this could be expected as parents and healthcare professionals might take a protective stance towards the child. However, the child might prefer to be protected in some situations and wants to share decision-making in other situations. In addition, some children prefer to leave the more ‘serious’ decisions to their parents and healthcare professionals, whereas other children prefer to share the decision [19, 35]. The views expressed by the participants seemed to confirm that mainly the non-treatment related decisions where shared, or the less ‘serious’ ones. Combined with the situation that the clinician ‘decides’ which information to share with a patient, the stage for SDM is not ideal [32, 36, 37].

The intention of the Dutch Pediatric Association was to potentially enhance the practice of SDM in pediatric palliative care and to do so by integrating SDM in the guideline PCFC. Therefore, recommendations on SDM were included in the guideline and the necessity of SDM was explained in a separate chapter. However, these SDM recommendations have not been integrated in the treatment related recommendations. The integration of SDM could be done e.g. by re-phrasing these recommendations to increase option awareness and/or to include patient preferences. Other possibilities are to structure the deliberation process and describe it more explicit in the guideline, and/or providing patient support tools [38]. These tools could be linked to a specific recommendation or to the guideline as a whole [5]. In the current format, the guideline might not encourage the interviewed pediatricians to involve patients/parents when talking about treatment choices which might inadvertently contribute to SDM only being used for non-treatment related decisions in children’s palliative care.

Participants were open to recommendations related to an SDM-approach to treatment decisions (i.e. structuring the options in a recommendation to increase option awareness), as was shown when we discussed the modified recommendation on pain relief. More than half preferred the ‘SDM’-recommendation because it can help open the discussion, it shows underlying arguments for different treatments, such as pros and cons, and it enables patients and/or parents to choose. Recommendations such as these are preferable according to the Institute of Medicine (IoM). In its report ‘Clinical practice guidelines we can trust’, IoM suggests refraining from so-called blanket recommendations: a recommendation for all patients to choose one particular treatment, irrespective of the patient’s characteristics, preferences and values. IoM recommends to describe the options and trade-offs in a recommendation encouraging SDM, as its respects the individual choice. In this way guidelines, according to IoM, become tools for patient engagement and activation [10]. The last couple of years more CPGs are being developed in which recommendations address trade-offs and mention more than one option [6, 39–42].

Another strategy to adapt guidelines so that they could enhance SDM is imbedding patient support tools – such as PDAs – in CPGs. The majority of participants thought that a guideline (recommendation) accompanied by a PDA would be beneficial to engaging patients and sharing the decision-making. Systematic reviews have shown that the use of PDAs supports patients to engage in deciding about their care [43]. Our interviews suggest that the use of PDAs could also help pediatricians to check if they have covered all the topics (PDA as a checklist) and address issues they would normally forget or not consider important. Another benefit of PDAs mentioned by pediatricians was that patients can take the PDA home and reread it. Patients often do not remember everything when talking with the clinician, and by taking it home they can weigh the pros and cons in a more comfortable and less time-pressured setting. PDAs can also help patients to ask questions, as patients not always dare to ask their pediatrician everything [44, 45]. The pediatricians who were not convinced of the benefit of a PDA claimed that patients did not want to use them [8]. Research has shown that patients do want to be involved in (deciding about) their care, and need to be involved [31, 46, 47] including children and adolescents (and their parents) [48–53]. Another reason why some pediatricians were not inclined to use a PDA was that they felt they know best what to advise based on their expertise, experience with the treatments and the patient sitting in front of them. This attitude is risky for three reasons; firstly, well-informed patients who actively participate in their care are more satisfied with their decisions [54]. Secondly, without using a PDA, the clinician will likely not be complete when providing information to the patient [36, 37]. And thirdly, it has also been shown that clinicians are not always correct in predicting what the patient wants [55, 56]. Moreover, the preferences of a clinician, from the perspective of being a patient herself or himself, often do not match with what they would recommend the patient [57]. Instead of projecting their own opinion on their patients, clinicians should ask what the patient prefers; not only when it concerns treatment decisions but also when it comes to being involved and deciding about their care.

Another important finding is that participants were worried about burdening parents with the responsibility of deciding on their child’s end-of-life care if they engaged them in SDM. However, instead of assuming that parents do not want to be burdened with this responsibility, discussing this with the parents, and/or child, acknowledging their autonomy in the decision-making process, seems to be justified and fitting with SDM. Some research has shown that parents want to be the ultimate decision maker for their child [58], and children value autonomous decision-making, without excluding their parents [51, 59]. In the interviews the pediatricians used the words responsibility and sharing the decision-making interchangeably. It appears that the pediatricians are not aware that even when the decision-making is shared with the child and/or parents, they have the final responsibility for the decision. This unclarity might make pediatricians wary of SDM when it comes to sharing decisions.

Regarding the comments made by the pediatricians that SDM is a skill, and that the recommendations in the PCFC guideline mainly addressed the *what* and not the *how* of SDM, the guideline could be enriched with examples of SDM in the pediatric palliative care context. For example, Van der Weijden et al. suggest using a vignette describing how a patient and a healthcare provider discuss the options to reach a shared decision, or could provide scripts modelling SDM-language. However, enhancement of the practice of shared decision-making requires more than an isolated guideline which integrates SDM, especially when it comes to skills and attitude. It needs to be imbedded in an overall SDM contextualized effort for SDM to become common practice. Healthcare professionals could be trained, receive feedback on SDM performance, be facilitated in an SDM approach, work in organizations which advocate SDM and where there is senior-level buy-in of engaging patients in their care and sharing the decision-making with them [9, 32].

### Limitations and strengths

Limitations of this study are that the findings are limited to the 15 participants and the university medical centers (3) they work at. Even though the participating centers were considered forerunners of SDM, it does not mean that other pediatricians in other medical centers have the same opinions. However, data saturation occurred after the eight interviews. Another limitation was the timing of the interviews; in hindsight, the interviews might have taken place too early as the guideline PCFC had not been fully implemented yet. Which meant that most of the participants had not used the guideline yet, and some had only heard of it. This was countered by showing the participants the guideline and by walking through the recommendations on SDM. Key strengths were that we conducted the research with the developers of the PCFC guideline themselves (EV, LK) and that participants were very forthcoming and open during the interviews as they were being interviewed by a doctor to be. We also carried out a member check on the interview transcripts.

## Conclusions

The interviews showed that most of the participants thought that CPGs in general potentially could enhance SDM. However, integrating SDM into a guideline seems not to be an easy feat, and guideline developers have to walk a tightrope on how to formulate recommendations on SDM. They have to avoid stating the obvious because it might offend and alienate the pediatrician. At the same time, they have to provide more detail on how to practice SDM. Furthermore, developers could consider formulating more ‘open’ recommendations. Especially in case of preference sensitive choices, the recommendation should describe (treatment) trade-offs and (treatment) alternatives and provide more detailed guidance. Another consideration is to provide tools amalgamated with specific guideline recommendations that enhance SDM, such as PDAs.

## Supplementary information

**Additional file 1.** Modified guideline recommendation on pain relief.

**Additional file 2.** Interview guide.

**Additional file 3.** Code book final.

## Data Availability

Part of the data generated or analyzed during this study are included in this published article [and its supplementary information files]. NVivo datasets used and/or analyzed during the current study are available from the corresponding author on reasonable request.

## Abbreviations

CPG: Clinical practice guideline
IoM: Institute of Medicine
PCFC: Palliative care for children (guideline)
PDA: Patient decision aid
SDM: Shared decision-making

## References

[1] Centraal Bureau voor de Statistiek (2012). StatLine. Den Haag.

[2] Molenkamp CM, Hamers JPH, Courtens AM (2005). Een exploratieve studie naar zorgbehoeften, aanbod, knelpunten en mogelijke oplossingen (palliative care for children; Taylor-made approach needed. An exploratory study into care needs, care options, bottle necks and possible solutions). Palliatieve zorg voor kinderen: maatwerk vereist.

[3] Guideline Palliative care for children [https://www.nvk.nl/Kwaliteit/Richtlijnen-overzicht/Details/articleType/ArticleView/articleId/894].

[4] Karnieli-Miller O, Eisikovits Z (2009). Physician as partner or salesman? Shared decision-making in real-time encounters. Soc Sci Med.

[5] van der Weijden T, Pieterse AH, Koelewijn-van Loon MS, Knaapen L, Legare F, Boivin A, Burgers JS, Stiggelbout AM, Faber M, Elwyn G (2013). How can clinical practice guidelines be adapted to facilitate shared decision making? A qualitative key-informant study. BMJ Qual Saf.

[6] Armstrong MJ, Gronseth GS (2018). Approach to assessing and using clinical practice guidelines. Neurol Clin Pract.

[7] Gravel K, Legare F, Graham ID (2006). Barriers and facilitators to implementing shared decision-making in clinical practice: a systematic review of health professionals’ perceptions. Implement Sci.

[8] Joseph-Williams N, Lloyd A, Edwards A, Stobbart L, Tomson D, Macphail S, Dodd C, Brain K, Elwyn G, Thomson R (2017). Implementing shared decision making in the NHS: lessons from the MAGIC programme. Bmj.

[9] van der Weijden T, Post H, Brand PLP, van Veenendaal H, Drenthen T, van Mierlo LA, Stalmeier P, Damman OC, Stiggelbout A (2017). Shared decision making, a buzz-word in the Netherlands, the pace quickens towards nationwide implementation. Z Evid Fortbild Qual Gesundhwes.

[10] Inistitute of Medicine (2011). Clinical Practice Guidelines We Can Trust.

[11] Galla JH (2000). Clinical practice guideline on shared decision-making in the appropriate initiation of and withdrawal from dialysis. The renal physicians association and the American Society of Nephrology. J Am Soc Nephrol.

[12] Moss AH (2001). Shared decision-making in dialysis: the new RPA/ASN guideline on appropriate initiation and withdrawal of treatment. Am J Kidney Dis.

[13] Patel SS, Holley JL (2008). Withholding and withdrawing dialysis in the intensive care unit: benefits derived from consulting the renal physicians association/american society of nephrology clinical practice guideline, shared decision-making in the appropriate initiation of and withdrawal from dialysis. Clin J Am Soc Nephrol.

[14] Feudtner C (2007). Collaborative communication in pediatric palliative care: a foundation for problem-solving and decision-making. Pediatr Clin N Am.

[15] Wet op de geneeskundige behandelovereenkomst (Medical Treatment Contracts Act) Issued by Ministry of Justice and Security. The Hague: Ministerie van Justitie en Veiligheid (Ministry of Justice and Security); 2006.

[16] Hsiao JL, Evan EE, Zeltzer LK (2007). Parent and child perspectives on physician communication in pediatric palliative care. Palliat Support Care.

[17] Zwaanswijk M, Tates K, van Dulmen S, Hoogerbrugge PM, Kamps WA, Bensing JM (2007). Young patients’, parents’, and survivors’ communication preferences in paediatric oncology: results of online focus groups. BMC Pediatr.

[18] Coyne I (2008). Children's participation in consultations and decision-making at health service level: a review of the literature. Int J Nurs Stud.

[19] Coyne I, Gallagher P (2011). Participation in communication and decision-making: children and young people's experiences in a hospital setting. J Clin Nurs.

[20] de Vos MA, van der Heide A, Maurice-Stam H, OF B, Plotz FB, Schouten-van Meeteren AY, Willems DL, Heymans HS, Bos AP (2011). The process of end-of-life decision-making in pediatrics: a national survey in the Netherlands. Pediatrics.

[21] O’Brien BC, Harris IB, Beckman TJ, Reed DA, Cook DA (2014). Standards for reporting qualitative research: a synthesis of recommendations. Acad Med.

[22] Tong A, Sainsbury P, Craig J (2007). Consolidated criteria for reporting qualitative research (COREQ): a 32-item checklist for interviews and focus groups. Int J Qual Health Care.

[23] Creswell JW (2013). Qualitative inquiry and research design; choosing among five approaches.

[24] Lester S (1999). An introduction to phenomenological research.

[25] Evers J (2007). Kwalitatief interviewen: kunst én kunde (qualitative interviewing; art and practice).

[26] Decision aid Bone infection [http://www.cincinnatichildrens.org/service/j/anderson-center/evidence-based-care/decision-aids/].

[27] Care CftQoH (2012). Richtlijn voor richtlijnen: 20 criteria voor het ontwikkelen en implementeren van een klinische richtlijn.

[28] Guyatt GH, Oxman AD, Kunz R, Falck-Ytter Y, Vist GE, Liberati A, Schunemann HJ (2008). Group GW: Going from evidence to recommendations. Bmj.

[29] Couet N, Desroches S, Robitaille H, Vaillancourt H, Leblanc A, Turcotte S, Elwyn G, Legare F (2015). Assessments of the extent to which health-care providers involve patients in decision making: a systematic review of studies using the OPTION instrument. Health Expect.

[30] Pieterse AH, Henselmans I, de Haes HC, Koning CC, Geijsen ED, Smets EM (2011). Shared decision making: prostate cancer patients’ appraisal of treatment alternatives and oncologists’ eliciting and responding behavior, an explorative study. Patient Educ Couns.

[31] Coulter A (2017). Shared decision making: everyone wants it, so why isn't it happening?. World Psychiatry.

[32] Facilitating shared decision-making with parents of chronically ill children [http://www.policylap.chop.edu/ (Accessed 14 May 2014)].

[33] Wiering BM, Noordman J, Tates K, Zwaanswijk M, Elwyn G, De Bont ES, Beishuizen A, Hoogerbrugge PM, Van Dulmen S (2016). Sharing decisions during diagnostic consultations; an observational study in pediatric oncology. Patient Educ Couns.

[34] Brom L, De Snoo-Trimp JC, Onwuteaka-Philipsen BD, Widdershoven GA, Stiggelbout AM, Pasman HR (2017). Challenges in shared decision making in advanced cancer care: a qualitative longitudinal observational and interview study. Health Expect.

[35] Coyne I, Harder M (2011). Children's participation in decision-making: balancing protection with shared decision-making using a situational perspective. J Child Health Care.

[36] Dyer C (2015). Doctors should not cherry pick what information to give patients, court rules. Bmj.

[37] Snijders HS, Kunneman M, Bonsing BA, de Vries AC, Tollenaar RA, Pieterse AH, Stiggelbout AM (2014). Preoperative risk information and patient involvement in surgical treatment for rectal and sigmoid cancer. Color Dis.

[38] van der Weijden T, Dreesens D, Faber MJ, Bos N, Drenthen T, Maas I, Kersten S, Malanda U, van der Scheur S, Post H (2019). Developing quality criteria for patient-directed knowledge tools related to clinical practice guidelines. A development and consensus study. Health Expect.

[39] Eckman MH, Wise RE, Naylor K, Arduser L, Lip GY, Kissela B, Flaherty M, Kleindorfer D, Khan F, Schauer DP (2015). Developing an atrial fibrillation guideline support tool (AFGuST) for shared decision making. Curr Med Res Opin.

[40] Lumbosacraal radiculair syndrom (lumbosacral syndrome) [https://www.nhg.org/standaarden/volledig/nhg-standaard-lumbosacraal-radiculair-syndroom-lrs].

[41] Antimicrobial stewardship: systems and processes for effective antimicrobial medicine use [https://www.nice.org.uk/guidance/NG15]. 10.1093/jacamr/dlz025 PMC8210248 34222900

[42] Stokes T (2010). NICE clinical guidelines: involving patients, sharing decision-making, considering cost effectiveness. Huisarts Wetenschap.

[43] Stacey D, Legare F, Lewis K, Barry MJ, Bennett CL, Eden KB, Holmes-Rovner M, Llewellyn-Thomas H, Lyddiatt A, Thomson R (2017). Decision aids for people facing health treatment or screening decisions. Cochrane Database Syst Rev.

[44] Rodenbach RA, Brandes K, Fiscella K, Kravitz RL, Butow PN, Walczak A, Duberstein PR, Sullivan P, Hoh B, Xing G (2017). Promoting end-of-life discussions in advanced Cancer: effects of patient coaching and question prompt lists. J Clin Oncol.

[45] Sansoni J, Grootemaat P, Duncan C, Samsa P, Eagar K (2014). A systematic literature review on question prompt lists in health care (final report).

[46] Barry MJ, Edgman-Levitan S (2012). Shared decision making--pinnacle of patient-centered care. N Engl J Med.

[47] Charles C, Gafni A, Whelan T (1997). Shared decision-making in the medical encounter: what does it mean? (or it takes at least two to tango). Soc Sci Med.

[48] Hinds PS, Drew D, Oakes LL, Fouladi M, Spunt SL, Church C, Furman WL (2005). End-of-life care preferences of pediatric patients with cancer. J Clin Oncol.

[49] Lyon ME, McCabe MA, Patel KM, D’Angelo LJ (2004). What do adolescents want? An exploratory study regarding end-of-life decision-making. J Adolesc Health.

[50] Mack JW, Feudtner C, Hinds PS (2012). Communication and decision support for children with advanced Cancer and their families. American Society of Clinical Oncology educational book / ASCO American Society of Clinical Oncology Meeting.

[51] Pousset G, Bilsen J, De Wilde J, Benoit Y, Verlooy J, Bomans A, Deliens L, Mortier F (2009). Attitudes of adolescent cancer survivors toward end-of-life decisions for minors. Pediatrics.

[52] Sullivan J, Gillam L, Monagle P (2015). Parents and end-of-life decision-making for their child: roles and responsibilities. BMJ Support Palliat Care.

[53] Coyne I, Amory A, Kiernan G, Gibson F (2014). Children’s participation in shared decision-making: children, adolescents, parents and healthcare professionals’ perspectives and experiences. Eur J Oncol Nurs.

[54] Gochman DS, Bonham GS (1988). Physicians and the hospice decision: awareness, discussion, reasons and satisfaction. Hosp J.

[55] Stalmeier PF, van Tol-Geerdink JJ, van Lin EN, Schimmel E, Huizenga H, van Daal WA, Leer JW (2007). Doctors’ and patients’ preferences for participation and treatment in curative prostate cancer radiotherapy. J Clin Oncol.

[56] Mulley AG, Trimble C, Elwyn G (2012). Stop the silent misdiagnosis: patients’ preferences matter. Bmj.

[57] Ubel PA, Angott AM, Zikmund-Fisher BJ (2011). Physicians recommend different treatments for patients than they would choose for themselves. Arch Intern Med.

[58] Sullivan J, Monagle P, Gillam L (2014). What parents want from doctors in end-of-life decision-making for children. Arch Dis Child.

[59] Young B, Dixon-Woods M, Windridge KC, Heney D (2003). Managing communication with young people who have a potentially life threatening chronic illness: qualitative study of patients and parents. BMJ.

