# Supplementary material for: The clinical practice guideline palliative care for children and other strategies to enhance shared decision-making in pediatric palliative care; pediatricians’ critical reflections
Source: BMC Pediatr. 2019 Nov 29;19:467. doi: 10.1186/s12887-019-1849-0 (PMC6883587; doi:10.1186/s12887-019-1849-0)
Supplement: Supplementary file 1 — Additional file 1. Modified guideline recommendation on pain relief. [file 12887_2019_1849_MOESM1_ESM.docx]

Additional file 1: Modified guideline recommendation on pain relief

| Original recommendation CPG Palliative care for children | Modified multi-option guideline recommendation |
| --- | --- |
| The first choice in pain relief in the palliative phase is product X with dose A. | The first choice in pain relief in the palliative phase is product X with dosage A. After deliberation with the child/parents, you can decide on a lower dosage B or C.  Considerations:   - Most of the children/parents want to be informed on the side-effects, especially the side-effect drowsiness. (ref. xxxx) - For some children/parents the benefits of pain relief do not outweigh the side-effects (drowsiness of the child). (ref. xxyz) - Heterogeneity exists in the preferences of children/parents: 30% choses dose A; 50% choses dose B; and 20% choses dose C. (ref. xyzx) |
