# Supplementary material for: The clinical practice guideline palliative care for children and other strategies to enhance shared decision-making in pediatric palliative care; pediatricians’ critical reflections
Source: BMC Pediatr. 2019 Nov 29;19:467. doi: 10.1186/s12887-019-1849-0 (PMC6883587; doi:10.1186/s12887-019-1849-0)
Supplement: Supplementary file 2 — Additional file 2. Interview guide. [file 12887_2019_1849_MOESM2_ESM.docx]

**Additional file 2 - Interview guide**

NB: During the interview, the participants were shown a hard copy of the CPG Palliative care for children.

|  | **Questions** | **Primers, examples and reminders for interviewer** |
| --- | --- | --- |
| 1  1.1  1.2  1.3 | Guideline (< 5 minutes)  Can you briefly explain what you consider clinical practice guidelines to be?  Do you apply them? When/how do you apply them?  Do you find the guidelines beneficial to patient contacts? Why/why not and how? | *a.k.a. clinical practice guideline*  *National guidelines, local guidelines, protocols, other? Does the answer clearly reveal the interviewee's opinion?* |
| 2  2.1  2.2  2.3 | Decision-making  Are you familiar with the practice of shared decision-making (SDM)?   - If yes: what do you consider SDM to be? - If no: read the definition out loud and ask for a reaction.   What is your opinion of SDM?  Having discussed this, do you believe guidelines offer possibilities for improving shared decision-making among doctors and between doctors and patients? What I mean by this is that a doctor who uses guidelines during interviews anyway feels more stimulated, supported or assisted to actively use guidelines. | … *remain quiet, leave room for explanations, if only yes or no then…*  *If the interviewee gives his/her opinion straight away, follow up by asking what they mean*  *… If the interviewee gives another description, let them read the working definition used in the study and ask for the interviewee's reaction (Can I summarize your answer as …, for this study we used this definition – what is your reaction?)*  *If yes: how and why?* |
| 3  3.1  3.2  3.3  3.4  (3.5)  3.6 | Guideline Palliative care for children  The national guideline Palliative care for children was introduced in the summer of 2013.  Are you familiar with this guideline?  How?   - (If yes: can you summarize your opinion of this guideline in two sentences? - What did you find most striking about the guideline? - Do you ever use the guideline?   - If yes: which part of the guideline do you use and/or in which situation/when?     - When did you last use the guideline?   - If not: why not?   An innovation introduced by this guideline is a set of recommendations for the decision-making and organization process.  (Are you familiar with these? (familiar with these elements, which were not mentioned under 3.1))   - If yes: do you use them? Why or why not? - What is your opinion of the inclusion of this type of recommendations in guidelines?   We have a number of other examples of how SDM can be improved:   - Two decision aids: (1) anticoagulant comparison and (2) step-by-step plan for bone infection - What is your opinion?   We have also made an SDM recommendation of our own.   - What is your opinion?   Do you have a preference? And why?  Casting a quick glance over a printout of the guideline, what is your first reaction?  Form, usefulness, size, colors, number of recommendations, volume, availability in digital format  Earlier in the interview, we discussed whether guidelines could contribute to shared decision-making. Your answer at the time:… Did you find this guideline (if known to the interviewee) supportive?  If yes: in what way?  Can you think of other aids that would have been helpful in this respect?  Which aids do you feel could help patients and their parents become more involved in the healthcare process?  The final question I'd like to ask you about the guideline Palliative care for children is: has special attention been paid to this guideline on your ward?   - If yes: how was the guideline implemented on your ward?   - By whom?   - How?   - When? (> 1x)   - What was your opinion? - If no: do you know why not? | - *Seen?* - *Read?* - *Developed?* - *Implemented?*   *Summary, recommendations, decision chart, brochure for parents, indicators… Situations/moments: prior to, during or after the discussion/multidisciplinary meeting/consultation, uncertainty, multiple options, no consensus…*  *Tools/training:*  *- Summary chart, flow chart, app, clinical path, decision aid, EPR link*  *- Words such as discuss, consult, ask the opinion of the parents/child, acknowledge their viewpoint*  *- Training, focus group, video (training),*  *trial implementation, peer review* |
| 4  4.1  4.2  4.3  4.4  4.5  4.6 | Decision-making in practice  Having concluded the theory part of this questionnaire, I'd now like to ask you about your experiences of decision-making in practice.  Discussion with the patient: can you name a few aspects of the consultation process that you would pay special attention to in order to assess whether colleagues/medical interns are using joint decision-making in a palliative setting?  Can you describe a case where the joint decision-making process ran smoothly?  And a case where it did not? And why?  Can you describe a palliative case to illustrate the decision-making process and the parties involved?  Patient file: do you record agreements/decisions you have made with the patients regarding joint decision-making in the patient file?   - If yes: how? - Were you aware that the guideline contains recommendations for this?   Multidisciplinary meeting: during multidisciplinary consultations, you discuss patient cases with your colleagues. How do you use the multidisciplinary consultations to discuss cases where there is not one single obvious treatment, but several options?  This study is partially aimed at promoting the implementation of the guideline, as well as at improving the guideline itself and suggesting potential aids. We discussed this earlier in the interview. We have now discussed the guideline in practice.   - Do you have any other suggestions as to how to improve the guideline following these discussions? - Or as to promoting its implementation? Who should deal with this at the hospital? - How about other aids that could help you implement joint decision-making in discussions? | *Tools/training:*  *- Summary chart, flow chart, app, clinical path, decision aid, EPR link…*  *- Training, focus group, video (training), trial implementation, peer review…* |
| 5  5.1 | Final questions  We are approaching the end of the interview.  Do you have any further questions or comments?  Thank you for your cooperation. I will now stop the tape.  (Evaluation after the tape has been stopped: what went well and what could have gone better during the interview?) |  |
