# Supplementary material for: The clinical practice guideline palliative care for children and other strategies to enhance shared decision-making in pediatric palliative care; pediatricians’ critical reflections
Source: BMC Pediatr. 2019 Nov 29;19:467. doi: 10.1186/s12887-019-1849-0 (PMC6883587; doi:10.1186/s12887-019-1849-0)
Supplement: Supplementary file 3 — Additional file 3. Code book final. [file 12887_2019_1849_MOESM3_ESM.docx]

**Code book final - English**

**Clinical practice guideline (CPG)**

CPG: description

- CPG definition
- Scope of CPG concept (= broad concept)
- Multidisciplinary CPG
- CPG synonym/related term (reference point, reference work)
- Difference between protocol/CPG

CPG: qualification

- CPG qualification: evidence-based
- CPG qualification: legibility (illegible)
- CPG qualification: size (too big)
- CPG qualification: everyday practice

CPG: use/practice

- Use
- Level of care being protocolised
- Physical CPG use (opening the CPG)
- Level of CPG use
- (condition of CPG use) = manageability
- CPG as a checklist
- CPG searchability
- CPG supports decision-making
- CPG 🡪 scope for deviation / non-compliance
- Care ≠ 100% evidence-based
- Working according to CPG
- Care covered by CPG
- Importance of CPG to parents/patients
- Contribution of CPG to patient contact
- Difference between CPG in theory and practice
- CPG and doctor's legal position

CPG: knowledge

- CPG internationalization (knowledge)
- Limited knowledge of professionals (e.g. due to specialization)
- CPG makes knowledge accessible
- CPG if knowledge area is not part of daily routine
- CPG as counterweight to ‘random’ information
- Knowledge-sharing in a profession not practiced by a lot
- Guideline = knowledge instrument

CPG: uniformization

- Importance of uniformization
- Uniformization (due to working with protocols/guidelines) =
- Inter…variation (professional, center, …)

CPG: development and implementation

- CPG development process
- Purpose of international cooperation (e.g. in CPG development)
- CPG and local implementation
- Difference between CPG and local practice

**Shared decision making (SDM)**

SDM: description

- SDM definition
- SDM definition unknown
- SDM = modern healthcare
- SDM ≠ paternalism
- SDM = Multidisciplinary meeting (MDM)
- SDM = freedom of choice
- Care = SDM

SDM: information

- SDM = inform patient/parents

SDM: option

- SDM = options incl. pros and cons
- SDM = optional talk after MDM (with parents)
- SDM = point out consequences of preferences
- SDM = explain possibilities
- SDM = steer towards a decision
- SDM ≠ steer towards a decision

SDM: decision

- SDM = shared decision-making (patient + doctor)
- SDM = share the decision-making
- SDM = together
- SDM = parents/patient decide
- SDM = parents agree with chosen policy
- SDM = involving the child in the decision-making
- SDM = doctor decides in case of superior option
- SDM = parents decide in case of grey area
- SDM = discuss consequences of decision
- SDM = make agreements
- SDM = seek consensus
- SDM = second opinion
- SDM = agreements in MDM
- SDM = live with decision
- SDM = patient/parents take no medical decisions
- SDM = shared decision-making not compulsory

SDM and consult/talk

- SDM = importance of interaction
- SDM = conversation technique
- SDM = room for acceptance
- SDM = transparency
- SDM = gather support
- SDM = keep in touch/show (and maintain) interest
- SDM = room for discussion
- SDM = share precautions
- SDM = check that info/discussed topic has been understood
- SDM = give room if parents disagree with policy
- SDM = create openness in first discussion
- Conversation techniques contribute to SDM
- SDM = importance of non-verbal communication
- Medical interns conduct no palliative care (PC) discussions
- Trap: think on behalf of parents/patient
- Trap: fall back on knowledge (instead of feeling)
- SDM = room for emotions
- Doctor knows what patient/parents want

SDM: role of doctor

- SDM = doctor advises
- SDM = doctor knows best
- SDM = responsibility lies with doctor
- SDM = attitude/competence
- SDM = gauge opinion of parents/ patient
- SDM = ask parents for their perception

SDM: role of patient/parents

- SDM = initiative with parents
- Role of patient/parents in SDM
- SDM contributes to patient's autonomy
- Should parents know everything (about CPG)
- SDM = stress of choice for patient/parents

SDM and MDC

- SDM = include parents' viewpoint in MDM

SDM and practice

- SDM in practice
- SDM = not easy in practice
- SDM differs per phase
- Differences in palliative care paths
- When to apply SDM
- How to apply SDM
- Limited choice in child oncology/IC/Neonatal care
- SDM = not every detail
- SDM = always involve parents/patient
- SDM = importance of parental involvement
- SDM = dependent on parents’ capability
- SDM depends on equality of knowledge
- SDM = dependent on type of parents
- SDM should be included in training (explanation: in order to equip doctors to implement/practice SDM, it should become part of their training)

SDM: assessment

- Importance of SDM
- SDM = must/always (ethical/principle)
- SDM = common sense
- Usefulness/purpose of SDM (compliance, mastery)

SDM: limitation

- SDM obstacles
- SDM and time
- SDM time as limitation
- Complexity of SDM practice (surrogate parents)
- SDM: guideline
- Need of CPG on SDM or an SDM CPG

SDM in CPG positive

- CPG can improve SDM

SDM in CPG negative

- CPG hampers SDM
- Little room for SDM in CPGs
- Doubts regarding effectiveness of SDM in CPG on SDM in practice
- SDM in CPG does not contribute to SDM attitude

SDM: choice of words in CPG

SDM case

- SDM case description positive

- SDM case description negative

SDM recommendation

- Preference for SDM recommendation

- Preference for short recommendation

- SDM recommendation leads to SDM

- Relation between SDM recommendation and CPG size

- Disadvantage of SDM recommendation

- Preference for type of recommendation depends on situation

**CPG Palliative care for children (PCfC)**

CPG PCfC: use

- Familiar with CPG PCfC

- Familiar with "entire" CPG PCfC
- Familiar with parts of CPG PCfC
- Familiar with existence of CPG PCfC (e.g. through cooperation)
- Not familiar with CPG content
- CPG PCfC use
- CPG PCfC not used
- CPG PCfC not read
- Intention to use CPG PCfC
- CPG PCfC as knowledge instrument
- Relation between CPG use/introduction and release date
- CPG PCfC as reference work
- Importance of acting in practice ≠ CPG
- Amount of clinician’s expertise in regard to need of using CPG
- Match between CPG PCfC and daily practice

CPG PCfC: assessment

- Positive existence of CPG PCfC
- CPG PCfC description
- CPG PCfC usefulness
- CPG PCfC usefulness negative
- CPG PCfC size
- CPG PCfC = illegible
- CPG PCfC not applicable to subspecialty
- CPG PCfC not usable
- CPG PCfC in digital format only is not useful
- Ease of CPG access/location
- CPG disadvantage: recommendation "states the obvious"
- CPG PCfC searchability
- Importance of CPG summary (usefulness/applicability thanks to summary)
- CPG PCfC invites reflection
- CPG PCfC provides support
- SDM language use in CPG PCfC
- CPG PCfC includes social context
- CPG PCfC is problem-oriented
- CPG PCfC color use is useful
- CPG PCfC in digital format only is fine
- Set of recommendations = summary
- Relation between topic size and CPG size
- Relation between CPG comprehensiveness and CPG size

GPF PCfC: Evidence-based medicine (EBM)

- CPg PCfC = EBM

CPG PCfC: Palliative Care (PC)

CPG PCfC: decision-making and organization

- Positive about CPG PCfC layout
- Negative about CPG PCfC layout
- Decision-making (belongs) in CPG
- Reach agreements regarding decision making (= CPG PCfC recommendation)
- Organization of care (belongs) in CPG

CPG PCfC implementation:

- App improves CPG PCfC implementation

**Decision Aid (DA)**

- Preference for oral advice rather than DA

- DA incomplete

- DA useful to patient/parents

- DA not useful to patient/parents

- DA is handy = useful to doctor?

- Comprehensiveness of DA information/checklist

- DA n/a or not required

**Implementation**

Local implementation

- No implementation on ward
- Implementation by ambassador/ward

Promotion of implementation

- Implementation thanks to publicity
- Implementation thanks to CPG PCfC evaluation
- CPG PCfC dissemination
- Usefulness of CPG summary for implementation
- Implementation thanks to peer review (= added value according to CPG)
- CPG must influence user action (= added value according to CPG)
- Implementation thanks to feedback of CPG compliance (= added value according to CPG)

Obstacles to implementation

- CPG delivery ≠ implementation
- Necessity of CPG implementation prior to application
- No active reminder to implement CPG PCfC

**File/notes**

Record keeping

- (Limited) record in file
- Record keeping by letter
- File: summary of discussion with parents
- Record keeping equals policy note keeping
- Parental opinion/decision in file
- Notes in file for transfer purposes
- Note in file regarding personal experience of patient/parents

CPG PCfC file recommendation

- CPG file recommendation (unknown)

**Multidisciplinary meeting (MDM)**

- No parents in MDM
- MDM options to be proposed to parents
